# Discordance Between p16-Expression and HPV-Status in Sinonasal Carcinoma: A Multicenter Retrospective Study

**DOI:** 10.3390/cancers17193135

**Published:** 2025-09-26

**Authors:** Nina Wenda, Henrike Barbara Zech, Marta Barde, Leoni Ramke, Anna Sophie Hoffmann, Till Clauditz, Sebastian Wagner, Jan Gosepath, Christian Stephan Betz

**Affiliations:** 1 Department of Otolaryngology, Head and Neck Surgery, Helios HSK Wiesbaden, 65199 Wiesbaden, Germany; marta.barde@helios-gesundheit.de (M.B.); jan.gosepath@helios-gesundheit.de (J.G.); 2 Department of Otolaryngology, Head and Neck Surgery, University Medical Center Hamburg-Eppendorf, 20251 Hamburg, Germany; h.zech@uke.de (H.B.Z.); l.ramke@uke.de (L.R.); c.betz@uke.de (C.S.B.); 3 Mildred-Scheel Cancer Career Center HaTriCS4, University Medical Center Hamburg-Eppendorf, 20251 Hamburg, Germany; 4 Department of Pathology, University Medical Center Hamburg-Eppendorf, 20246 Hamburg, Germany; t.clauditz@uke.de; 5 Department of Pathology, Helios HSK Wiesbaden, 65199 Wiesbaden, Germany; sebastian.wagner@helios-gesundheit.de

**Keywords:** sinonasal carcinoma, human papilloma virus, biomarker discordance, p16, HPV-PCR

## Abstract

Background/Objectives: Human papillomavirus (HPV) infection is a well-established risk factor for oropharyngeal squamous cell carcinoma (OPSCC), where p16 immunohistochemistry serves as a surrogate marker. However, the role of HPV in sinonasal squamous cell carcinoma (SNSCC) remains less defined, and the reliability of p16 as a standalone surrogate is under debate. This study aimed to assess the concordance between p16 expression and HPV-DNA status in SNSCC and characterize clinicopathologic features in HPV-associated cases. Methods: We retrospectively analyzed 111 SNSCC cases diagnosed between 2008 and 2024 at two German centers. p16 status was determined by immunohistochemistry using site-specific antibody protocols. HPV-DNA testing and genotyping were performed via PCR and reverse hybridization. Clinical and histopathological data were collected and compared between HPV-positive and -negative tumors. Results: HPV-DNA was detected in 31/111 cases (27.9%), with HPV16 and HPV33 (Site A) and HPV 16 and HPV18 (Site B) being the most frequent subtypes. Discordance between p16 and HPV-DNA status was observed in 29.7% of cases, with site-specific discordance rates of 44.6% and 14.5%. Patients with HPV-positive tumors were younger than their HPV-negative counterparts. Conclusions: Our findings underscore the limitations of p16 as a single surrogate marker for detecting HPV-associated sinonasal cancer. Future research on the role of HPV in sinonasal cancer should integrate complementary testing methods (like p16Ink4A immunohistochemistry and HPV DNA/mRNA analysis) and aim for test standardization.

## Simple Summary

Human papillomavirus (HPV) infection is known to be a risk factor of head and neck and especially throat cancer. A protein called p16 is often used as a detection marker for HPV-associated cancer. It is unclear whether cancers in the nose and sinuses (called sinonasal squamous cell carcinoma or SNSCC) are affected through HPV, and whether p16 is a reliable test for HPV in this disease. This study looked at 111 patients with SNSCC, diagnosed in two centers in Germany between 2008 and 2024. Tumors were tested for p16 and for HPV DNA. We found that about 28% of the tumors had HPV, mostly types 16 and 33. But in about 30% of cases, the p16 test results did not match the actual HPV DNA findings. This mismatch varied depending on the sites. Our findings suggest that p16 is insufficient as a standalone marker for detecting HPV-association. Method standardization and more precise testing is needed in cancers of the nasal cavity and paranasal sinus to the understanding of the biology of these rare and difficult-to-treat tumors.

## 1. Introduction

Human papillomavirus (HPV) has emerged as a relevant etiological factor in head and neck squamous cell carcinomas (HNSCC), particularly in oropharyngeal cancers, where HPV involvement is well established and associated with distinct clinical and prognostic implications [1]. In contrast, the role of HPV in sinonasal squamous cell carcinomas (SNSCC) has only recently entered the focus of scientific attention [2]. Although HPV DNA has been identified in a subset of sinonasal tumors, the understanding of its biological relevance and diagnostic utility in this anatomical region is still evolving.

Sinonasal malignancies are rare, accounting for less than 5% of all head and neck tumors, with an estimated incidence of 0.5 to 1.0 per 100,000 population. Among these, SNSCC represents the most common histologic subtype and comprises approximately 3% of all head and neck cancers [3]. Due to their rarity and histologic heterogeneity, the molecular underpinnings of these tumors remain incompletely understood.

p16 immunohistochemistry is widely used as a surrogate marker for transcriptionally active HPV in oropharyngeal squamous cell carcinoma (OPSCC). However, evidence from OPSCC has shown that p16 expression and HPV-DNA detection are not always concordant [4,5]. A subset of tumors exhibits p16 positivity despite lacking detectable HPV DNA, while others may be HPV-DNA-positive without concurrent p16 overexpression [6]. This bidirectional discordance is particularly relevant in light of ongoing efforts to develop tailored treatment strategies for HPV-associated tumors in the context of precision oncology [7]. As evidence for HPV involvement in SNSCC continues to grow, it becomes essential to investigate whether similar patterns of discordance occur in this rare and understudied tumor entity. In this context, the reliability of p16 as a standalone diagnostic marker warrants critical evaluation.

To address this question, we conducted a retrospective multicenter study including 111 patients with histologically confirmed SNSCC. All tumors were analyzed for p16 expression and HPV-DNA status, including subtyping, and correlated with clinical and demographic parameters.

## 2. Materials and Methods

### 2.1. Study Design and Patient Selection

This retrospective multicenter study included 111 patients diagnosed with histologically confirmed sinonasal squamous cell carcinoma (SNSCC) between 2008 and 2024.

Tumor samples were obtained from two German centers: one tertiary care center in South Germany (Site A) and one university Medical Center in Hamburg Eppendorf (UKE), North Germany (Site B), both located in metropolitan regions. Inclusion criteria comprised availability of formalin-fixed, paraffin-embedded (FFPE) tumor tissue, confirmed SNSCC histology, and accessible clinical and demographic data. Cases with incomplete tissue material or insufficient documentation were excluded.

### 2.2. Histopathological Evaluation and Immunohistochemistry

All FFPE tumor samples were re-evaluated by experienced head and neck pathologists to confirm histopathology diagnosis.

#### 2.2.1. Site A

p16 (CDKN2A, p16Ink4A) immunohistochemistry was performed using the monoclonal antibody clone 16P04 (Leica Biosystems, Nussloch, Germany; Cat. No. PA0201) following the manufacturer’s protocol. Strong and diffuse nuclear and cytoplasmic staining in ≥70% of tumor cells was considered p16-positive, in accordance with established guidelines for head and neck squamous cell carcinoma [8].

#### 2.2.2. Site B

Freshly cut tissue microarray (TMA) sections with a thickness of 3 μm were used for immunohistochemical analysis. All staining procedures were performed on the same day in a single experimental run. p16 immunostaining was carried out using a mouse monoclonal anti-p16 antibody (BD Biosciences, clone G175-405) at a dilution of 1:3600. Endogenous peroxidase activity was blocked by treating the slides with hydrogen peroxide (DAKO S2023) for 10 min. Antigen retrieval was performed by heating the slides in a citrate buffer (pH 7.8) in an autoclave for 5 min. The EnVision™ detection system (DAKO K5007) was used to visualize the immunostaining. Coherent with Site A, p16 expression was considered positive when ≥70% of tumor cells showed moderate to strong staining intensity [8]. For documentation, TMA slides were digitized using a whole-slide scanner (Aperio AT2, Leica) at 40× magnification.

### 2.3. HPV-DNA Detection and Subtyping

#### 2.3.1. Site A

HPV-DNA detection and genotyping were performed using the GenID^®^ HPV typing kit (GenID GmbH, Strassberg, Germany). Total DNA was extracted from epithelial tumor tissue, followed by polymerase chain reaction (PCR) and reverse hybridization for genotyping. The assay detects both low-risk HPV types (6, 11, 40, 42, 44, 54) and high-risk types (16, 18, 26, 31, 33, 35, 39, 45, 51, 52, 53, 56, 58, 59, 66, 67, 68, 69, 70, 73, 82, 85, 97). The probe for HPV35 is known to cross-react with HPV26. The test is validated for detecting clinically relevant viral loads down to 103 genome equivalents. Internal quality controls, including β-globin amplification, were used to ensure DNA integrity and adequacy.

#### 2.3.2. Site B

HPV genotyping was based on DNA sequencing of PCR products amplified using MY or MY/GP primers. The amplification products were purified using ExoSAP (USB^®^, Affymetrix, Santa Clara, CA, USA) and subsequently sequenced directly using fluorescent dye-labeled dideoxynucleotides and cycle sequencing. Sequencing was performed with the BigDye^®^ Terminator Cycle Sequencing Kit (Applied Biosystems, Waltham, MA, USA) on an ABI PRISM^®^ 3100 Genetic Analyzer (Applied Biosystems, Waltham, MA, USA).

The resulting sequences were analyzed using the BLAST (2.17.0) tool on the GenBank platform [9] for HPV genotype identification.

### 2.4. Data Collection and Statistical Analysis

Demographic data (age, sex) and clinical variables (tumor site, TNM stage at initial diagnosis) were extracted from medical records. Tumors were anatomically classified as arising from either the nasal cavity or paranasal sinuses. Descriptive statistics were used to summarize baseline characteristics. HPV-DNA status and p16 immunohistochemistry results were analyzed for concordance and discordance. Comparative analyses were performed between HPV-positive and HPV-negative subgroups regarding age, sex distribution, tumor site, histologic grade, and TNM classification. Continuous variables were compared using the unpaired *t*-test, and categorical variables were analyzed using the chi-squared test or Fisher’s exact test, as appropriate. *p*-values < 0.05 were considered statistically significant. Subgroup analyses were conducted for each study site to identify potential differences in testing results and discordance rates.

## 3. Results

### 3.1. Demographics and Tumor Characteristics

A total of 111 patients with histologically confirmed SNSCC were included in the study. Of these, 31 patients (28%) were classified as HPV-positive, while 80 patients (72%) were HPV-negative.

The mean age at diagnosis was 59.2 years (±14.4) in the HPV-positive group and 61.3 years (±13.0) in the HPV-negative group, with no statistically significant difference (*p* = 0.45). A male predominance was observed in both groups, with a male-to-female ratio of 2.88 in the HPV-positive and 1.67 in the HPV-negative cohort (*p* = 0.088).

The nasal cavity was the most common primary tumor site in both groups, accounting for 68% of HPV-positive and 78% of HPV-negative tumors. The maxillary sinus was the second most frequent site, observed in 10% of HPV-positive and 13% of HPV-negative cases (*p* = 1.00). Ethmoid sinus tumors were present in both groups, with 6 cases in the HPV-positive and 8 cases in the HPV-negative group. A single sphenoid sinus tumor was observed in the HPV-positive cohort, while none were reported in the HPV-negative group. The distribution of tumor sites did not differ significantly between groups (*p* = 0.93).

In terms of tumor grading, well to moderately differentiated tumors (G1–G2) were more common in both cohorts, representing 74% of HPV-positive and 69% of HPV-negative cases (*p* = 0.65). A history of inverted papilloma was documented in three patients, including two HPV-positive and one HPV-negative case.

Initial nodal involvement (N+) was observed in five patients: one in the HPV-positive group and four in the HPV-negative group. This difference was not statistically significant (*p* = 1.00; Fisher’s exact test). Distant metastases (M1) at diagnosis were also rare, affecting two patients in the HPV-negative cohort and none in the HPV-positive group (*p* = 1.00).

When grouping T-stage into early (T1–T2) and advanced (T3–T4) categories, 52% of HPV-positive tumors were T3–T4, compared to 34% of HPV-negative tumors. Although the difference did not reach statistical significance (*p* = 0.13), the early-to-advanced T-stage ratio differed notably: 0.94 in the HPV-positive group vs. 1.96 in the HPV-negative group, suggesting a trend toward more advanced disease at diagnosis in HPV-associated tumors. Table 1 summarizes the demographic and oncologic profile of the study population.

### 3.2. HPV-Subtype Analysis

**Figure 1:**
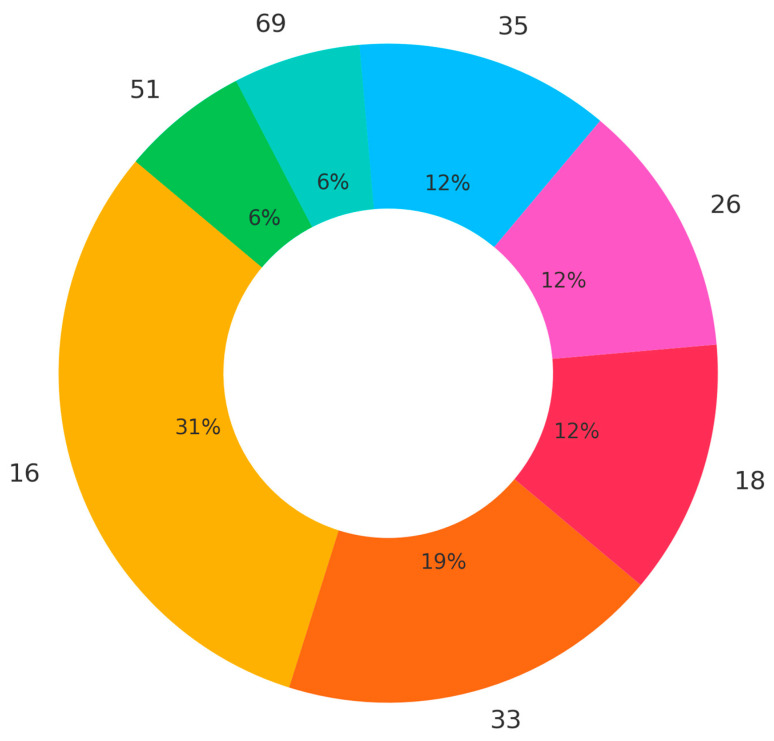
Distribution of HPV subtypes among the HPV-positive cases. Each slice of the pie chart represents a single subtype’s proportion.

Among the 31 HPV-DNA-positive tumors, a total of 38 high-risk HPV subtype entries were identified, as 8 tumors (25.8%) showed co-infection with two distinct high-risk subtypes. The most frequently detected subtype was HPV 16 (n = 14), followed by HPV 33 (n = 10) and HPV 18 (n = 8) with differences between the two sites (Site A: HPV16 (n = 12), HPV33 (n = 10), and HPV 18 (n = 3), and Site B: HPV16 (n = 2) and HPV18 (n = 5). Less common subtypes included HPV 26 and HPV 35 (each n = 2), and HPV 51 and HPV 69 (each n = 1). No low-risk HPV types were detected in any of the tumors. Figure 1 displays the distribution of HPV subtypes.

### 3.3. Relation of p16 and HPV Status

In the overall cohort of 111 patients, concordance between p16 expression and HPV-DNA status was observed in 78 cases (70.3%), including 62 p16−/HPV− and 16 p16+/HPV+ tumors. Discordant findings were present in 33 cases (29.7%), with 18 tumors showing p16 positivity despite lacking HPV-DNA and 15 tumors testing positive for HPV-DNA in the absence of p16 expression.

**Figure 2:**
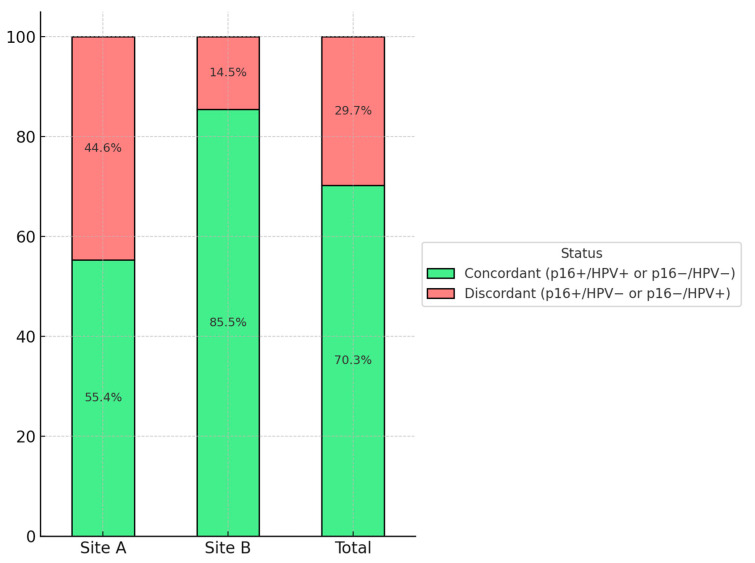
Percentage distribution of concordant and discordant p16 and HPV-DNA results, displayed for Site A, Site B, and the total cohort. Concordance refers to matching p16 and HPV-DNA status (either both positive or both negative). Discordance was defined as a mismatch between p16 immunoreactivity and HPV-DNA status.

Discordance rates varied markedly between sites. At Site A, 25 of 56 tumors (44.6%) showed discordant results, while Site B had only 8 of 55 discordant cases (14.5%). A visual summary of the concordance and discordance rates by site is presented in Figure 2. A detailed breakdown of p16 and HPV-DNA status is provided in Table 2.

## 4. Discussion

Approximately one in every 20 cancers diagnosed worldwide is attributable to HPV infection [10]. Within head and neck cancers, HPV is a well-established causative and prognostic factor for OPSCC, accounting for 70–80% of cases in the United States and about 60% in Germany [11,12]. Notably, HPV-positive OPSCC has now surpassed cervical cancer as the most common HPV-related malignancy in the USA [12]. Ongoing research aims to optimize treatment stratification for HPV-positive OPSCC and recently the first adjuvant de-escalation protocols were added into the National Comprehensive Cancer Network (NCCN) guideline [13]. While established in OPSCC, the role of HPV for sinonasal malignancies remains controversial. To determine whether HPV has prognostic significance and contributes to the oncogenic transformation of SNSCC, standardized and accurate diagnostic workflows are warranted. This study aims to evaluate the diagnostic utility of p16 immunohistochemistry and HPV PCR—standard methods for detecting HPV involvement in oropharyngeal cancer—in the context of SNSCC. Recent studies have provided evidence for the potential prognostic relevance of HPV-associated sinonasal squamous cell carcinoma (SNSCC) [14]. HPV-positive SNSCC has been shown to exhibit mutational profiles similar to those observed in HPV-driven cervical and oropharyngeal squamous cell carcinomas, supporting a biologically active role for HPV in SNSCC tumorigenesis rather than a passive “bystander” presence [15].

### 4.1. Relation of p16 and HPV Status

In OPSCC, the clinical standard for determining HPV association involves a two-step approach: initial detection of the surrogate marker p16 by immunohistochemistry, followed by HPV DNA testing—typically via polymerase chain reaction (PCR)—in p16-positive cases to confirm the presence and type of HPV. In this study, we observed a mismatch rate of nearly 30% between p16 immunohistochemistry and HPV-PCR across two independent SNSCC cohorts from metropolitan regions in Germany, each using different detection protocols. Notably, both discordance types—p16-positive/HPV-negative (16.2%) and p16-negative/HPV-positive (13.5%)—were present. These findings raise serious concerns about the reliability of p16 as a standalone surrogate marker for HPV association in SNSCC.

Discordance between p16 expression and HPV-DNA detection has also been reported in OPSCC, albeit less frequently. The largest multicenter, multinational retrospective analysis to date—including 7654 OPSCC patients—highlighted this issue: 3560 patients (46.5%) were p16–/HPV− (double negative), 3390 (44.3%) were p16+/HPV+ (double positive), while 415 (5.4%) were p16+/HPV− and 289 (3.8%) were p16−/HPV+. In other words, among the 3805 p16-positive patients, 415 (10.9%) showed no evidence of HPV; among the 3849 p16-negative patients, 289 (7.5%) were HPV-positive. This discordance was relevant for prognosis: 5-year disease-free survival differed markedly between subgroups—84.3% (95% CI: 82.9–85.7) in p16+/HPV+, 60.8% (95% CI: 58.8–62.9) in p16–/HPV−, 71.1% (95% CI: 64.7–78.2) in p16−/HPV+, and 67.9% (95% CI: 62.5–73.7) in p16+/HPV− cases [7]. In SNSCC, a p16/HPV-DNA discordance is less explored. The few studies with modest sample sizes show considerable variation depending on geographic region and methodology (see Table 3).

Reported discordance rates range from 0% to 16.7% for both p16-positive/HPV-negative and p16-negative/HPV-positive cases (see Table 3). HPV-DNA analysis in p16-negative SNSCC is rarely performed, even though our data suggest that p16-negative/HPV-positive status is at least as often as p16-positive/HPV-negative status. In our multi-center cohort, there was a notable discrepancy in HPV association and discordance rates between the centers in North and South Germany (14.5% vs. 44.6%). While differing methodological factors cannot be entirely ruled out, the consistency of testing protocols within each center argues against purely technical causes. Noteworthy and in line with this data, HPV distribution in oropharyngeal cancer also shows geographic variation [11]. Discordance rates of p16 and HPV-PCR- status are variable in different regions of Germany, e.g., in Cologne, Kiel and Giessen with 1.9%, 5.4%, 4.7% (p16−/HPV+) and 8.2%, 5.6% and 8.7% (p16+/HPV−) [7].

Higher p16/HPV-discordances in SNSCC compared to OPSCC could be due to site-specific differences in HPV-dependent downstream molecular pathways. As an example, the malignant transformation of inverted papilloma to SNSCC is often associated with HPV− infection and frequent loss of p16 expression [15]. In OPSCC, it could be shown that p16 induction relies critically on functional inactivation of the retinoblastoma (Rb) pathway by the HPV E7 oncoprotein. Absent p16 expression in HPV-positive SNSCC may result from transcriptionally inactive HPV infections or genetic or epigenetic alterations affecting the Rb pathway in SNSCC [8,21,22]. Overexpressed p16 in HPV-negative tumors, however, could be a result of oncogene-driven RB1 gene deletion or a mutation with loss of IHC Rb protein expression [8].

To sum it up: Our data showed that p16 alone should not be used as a stand-alone surrogative marker for HPV-driven oncogenesis in SNSCC.

### 4.2. Demographics and Tumor Characteristics

Previous analyses reported specific demographic characteristics in patients with HPV-positive SNSCC in comparison to their HPV-negative counterparts. They are significantly younger and more frequently male [23]. In our cohort, we observed a similar trend: the HPV-positive group had a lower mean age and a higher male-to-female ratio. Although neither difference reached statistical significance, the direction of these associations aligns with prior findings and suggests that HPV-related tumorigenesis in the sinonasal tract may affect a younger, predominantly male population. In our cohort, we observed a trend toward more advanced T-stage at diagnosis among HPV-positive SNSCC cases, with a lower early-to-advanced T-stage ratio (0.94) compared to HPV-negative cases (1.96), although this difference did not reach statistical significance (*p* = 0.13).

It remains a matter of debate whether HPV preferentially affects specific sinonasal subsites. Some studies have reported a higher prevalence of HPV in tumors arising from the nasal cavity compared to those originating in the paranasal sinuses [24], while this was not seen in others [20,21]. In our cohort, there were no site-specific manifestations in HPV-positive and –negative SNSCC, with more than two third of patients suffering from a tumor from the nasal cavity, in both groups.

### 4.3. HPV Subtypes

HPV-16 is the most oncogenic subtype and accounts for the overwhelming majority—over 90%—of HPV-associated HNSCC, particularly in the oropharynx. Other high-risk subtypes such as HPV-18, 31, 33, and 52 have been detected less frequently and are considered to play a minor role in most HNSCC cases [25]. However, HPV-33 has gained attention for its strong association with the recently described entity of HPV-related multiphenotypic sinonasal carcinoma (HMSC), which exhibits distinct histological and molecular features [26].

In our study, although HPV-16 was the most commonly detected individual subtype, it accounted for only 31% of HPV-positive tumors—substantially lower than the proportion typically seen in oropharyngeal cancers. HPV-33 was the second most frequent subtype and found in 10 cases, including several co-infections. Notably, we observed a relatively high proportion of tumors harboring multiple high-risk HPV subtypes, including dual infections involving HPV-16 and HPV-33 or HPV-18, a phenomenon which has also been described before [27]. These findings underscore the molecular heterogeneity of HPV-associated SNSCC and suggest that non-HPV16 high-risk subtypes may play a more prominent role in the sinonasal tract compared to the oropharynx.

Whether this reflects biological differences, anatomical tropism, or evolving histological subtypes such as HMSC warrants further investigation. Given the different results from Site A and B, the prevalence of different subtypes appears to be highly dependent on geography.

### 4.4. Limitations

This study has several limitations. First, due to its retrospective design, it is inherently prone to biases such as incomplete documentation. The lack of a centralized pathological review may have introduced interobserver variability, particularly in p16 immunohistochemistry interpretation. The sample size, although the first German and one of the larger cohorts in this rare tumor entity, remains limited—especially for subgroup analyses such as HPV subtype distribution or co-infection rates—reducing statistical power and generalizability. Furthermore, due to the retrospective multicenter setting, the application of tumor staging systems could not be consistently verified, particularly with respect to the distinct classification systems for maxillary versus other paranasal sinus tumors, potentially impacting T-stage grouping.

An additional limitation of our study is the use of different methodologies for p16 immunohistochemistry and HPV-DNA testing at the two participating centers. This heterogeneity resulted in markedly different discordance rates and complicates direct comparison between cohorts. However, such methodological variability also reflects the real-world situation: at present, testing protocols for p16 and HPV-DNA are not standardized across centers in Germany or internationally, which poses a significant challenge for consistent interpretation of HPV status in SNSCC. Ideally, uniform testing strategies should be applied to all patients, but achieving such harmonization will likely require considerable time and resources. Future projects are planned to perform comparative analyses of the same tumor samples with different technologies to better quantify methodological influences.

### 4.5. Clinical Implications

The observed discordance between p16 expression and HPV-DNA status in SNSCC highlights a critical need for improved diagnostic precision. Relying solely on p16 immunohistochemistry may lead to misclassification of HPV status, which in turn could result in inaccurate prognostic assumptions or inappropriate inclusion of patients in de-escalation or personalized therapy trials. As interest in individualized treatment regimens for HPV-associated head and neck cancers continues to grow, particularly for tumors with a more favorable prognosis, the importance of accurate biomarker-based stratification becomes increasingly evident. Incorporating confirmatory testing such as HPV-DNA or RNA-based assays into routine diagnostic workflows for SNSCC may help ensure that patients are appropriately categorized and managed.

The prognostic relevance of HPV status in SNSCC remains uncertain due to the rarity of the disease and the limited outcome data available. Nevertheless, the first single-center studies suggest a possible impact [2], and ongoing multicenter collaborations are expected to provide more definitive evidence. Based on the first clinical and preclinical data [28,29], it appears likely that HPV status also carries prognostic significance in SNSCC.

## 5. Conclusions

In this multicenter study, we provide the first German and one of the largest analyses to date of comprehensive p16 expression and HPV-DNA analysis in SNSCC, highlighting a substantial discordance between these two diagnostic markers. Our findings support the growing consensus that p16 is insufficient as a standalone surrogate for HPV-driven oncogenesis in cancer of the sinonasal tract.

Our data underscore the need for more robust diagnostic algorithms incorporating both p16 immunohistochemistry and molecular HPV testing in this rare entity to deepen the biological understanding of this widely unexplored entity. Future prospective studies are warranted to elucidate the biological and clinical—especially prognostic—implications of HPV in SNSCC.

## Figures and Tables

**Table 1:** Demographics and tumor characteristics.

Characteristics	HPV+ (n = 31)	HPV− (n = 80)	*p*-Value
Mean age (yrs)	59.2	61.3	0.45
Sex ratio (M/F)	2.88	1.67	0.088
**Primary site**			0.93
Maxillary	3 (10%)	10 (12.5%)	1
Ethmoid	6 (19%)	8(10%)	
Sphenoid	1 (3%)	None	
Nasal cavity	21 (68%)	62 (78%)	
**Pathological features**			
G1-G2:G3-G4	23 (74%):8 (26%)	55 (69%):25 (31%)	0.65
History of inverted papilloma	2 (6%)	1 (1%)	
**TNM stage**			
T1-T2:T3-T4	15 (48%):16 (52%)	53 (66%):27 (34%)	0.13
N+	1 (3%)	4 (5%)	1
M+	None	2 (2.5%)	

**Table 2:** Site-specific distribution of p16 and HPV-DNA status and rate of discordance. The cohort was divided into two sites (Site A and Site B), and the number of p16-positive, HPV-positive, and discordant cases was determined for each. Discordance was defined as mismatch between p16 immunoreactivity and HPV-DNA status.

Site	Cases	p16+	p16−	HPV+	HPV−	Discordance Rate
A	56	27	29	24	32	44.6%
B	55	7	48	7	48	14.5%
Total	111	34	77	31	80	29.7%

**Table 3:** Reported discordance rates between p16 immunohistochemistry and HPV-DNA status in SNSCC across international cohorts.

Publication (Year)	Country	Patients (n)	Detection Method	p16+/ HPV+	p16−/ HPV−	p16+/ HPV−	p16−/ HPV+	HPV Subtypes
Hu et al., 2020 [16]	China (Eastern)	84 (de novo) 30 (inverted papilloma)	p16 IHC, HPV DNA PCR and genotyping	2 (2.4%) 1 (3.3%)	67 (79.8%) 23 (76.7%)	14 (16.7%) 1 (3.3%)	1 (1.2%) 5 (16.7%)	16; 18
Jiromaru et al., 2020 [8]	Japan	101	p16 IHC, HPV DNA ISH	9 (8.9%)	82 (81.2%)	6 (5.9%)	4 (4.0%)	16; 18
Hirakawa et al., 2023 [17]	Japan	79	p16 IHC, HPV DNA PCR and ISH	10 (12.6%)	69 (87.3%)	0 (0%)	0 (0%)	6; 18; 33; and 52
Cohen et al., 2020 [18]	USA/ Florida	47	p16 IHC, HPV RNA ISH	7 (14.9%)	32 (68.1%)	4 (8.5%)	4 (8.5%)	Not specified
Larque et al., 2014 [19]	Spain	70	p16 IHC, HPV RNA	14 (20%)	56 (80%)	0	0	16; 33
Tendron et al., 2022 [20]	France	59	p16 IHC, HPV RNA ISH (RNAscope) PCR	9 (15.3%)	43 (72.9%) (“doubtful positive”: 5)	2 (3.4%)	Not performed	16; 18; 33

## Data Availability

The raw data supporting the conclusions of this article will be made available by the authors on request.

## Abbreviations

The following abbreviations are used in this manuscript:

HMSC	HPV-related multiphenotypic sinonasal carcinoma
HNSCC	Head and neck squamous cell carcinoma
HPV	Human papillomavirus
IHC	Immunohistochemistry
ISH	In situ hybridization
NCCN	National Comprehensive Cancer Network
OPSCC	Oropharyngeal squamous cell carcinoma
PCR	Polymerase chain reaction
SNSCC	Sinonasal squamous cell carcinoma
TMA	Tissue microarray
